# Second Order Kinetic Modeling of Headspace Solid Phase Microextraction of Flavors Released from Selected Food Model Systems

**DOI:** 10.3390/molecules190913894

**Published:** 2014-09-04

**Authors:** Jiyuan Zhang, Mun-Wai Cheong, Bin Yu, Philip Curran, Weibiao Zhou

**Affiliations:** 1 Food Science and Technology Programme, c/o Department of Chemistry, National University of Singapore, 3 Science Drive 3, 117543, Singapore; 2 Agilent Technologies, 1 Yishun Avenue 7, 768923, Singapore; 3 Firmenich Asia Pte. Ltd., 10 Tuas West Road, 638377, Singapore; 4 National University of Singapore (Suzhou) Research Institute, 377 Lin Quan Street, Suzhou Industrial Park, Suzhou 215123, Jiang Su, China

**Keywords:** flavor release, HS-SPME, mathematical modeling, alcoholic beverages, chewing gum

## Abstract

The application of headspace-solid phase microextraction (HS-SPME) has been widely used in various fields as a simple and versatile method, yet challenging in quantification. In order to improve the reproducibility in quantification, a mathematical model with its root in psychological modeling and chemical reactor modeling was developed, describing the kinetic behavior of aroma active compounds extracted by SPME from two different food model systems, *i.e.*, a semi-solid food and a liquid food. The model accounted for both adsorption and release of the analytes from SPME fiber, which occurred simultaneously but were counter-directed. The model had four parameters and their estimated values were found to be more reproducible than the direct measurement of the compounds themselves by instrumental analysis. With the relative standard deviations (RSD) of each parameter less than 5% and root mean square error (RMSE) less than 0.15, the model was proved to be a robust one in estimating the release of a wide range of low molecular weight acetates at three environmental temperatures *i.e.*, 30, 40 and 60 °C. More insights of SPME behavior regarding the small molecule analytes were also obtained through the kinetic parameters and the model itself.

## 1. Introduction

Headspace analysis is defined as a vapor-phase extraction which is commonly used for sampling of volatile organic compounds from a non-volatile liquid or solid [1]. Among various techniques, headspace-solid phase microextraction (HS-SPME) is being valued as a simple and versatile method with the capability in measuring trace compounds with concentrations in the ppb to low ppm range, while some applications even reaching the ppt range [1,2]. Furthermore, the advancement of instrumentation has propelled HS-SPME into the mainstream of routine GC analysis [1]. Until now, HS-SPME has been widely applied across many disciplines, e.g. environmental analysis [3], in-field air quality tests [4], the petroleum industry [5], and biomedical analysis [6]. It has also been widely used in food industry for safety [7], flavor generation [8] and flavor profiling [9]. HS-SPME provides alternative approach over traditional extraction methods, such as steam distillation or direct solvent extraction that analyze the flavor profile from food matrices. HS-SPME could facilitate the understanding on flavors released from a food matrix into the headspace, which are responsible for the smell perceived by olfactory system. Nevertheless, it is important to note that the complexity of HS-SPME analysis usually rises when analyzing a mixture of volatile compounds, which could be further complicated by other factors such as matrix effect, the choices of fiber coatings, incubation temperature, extraction time and pressure [10].

HS-SPME is an extraction process where analytes are in a dynamic equilibrium among the three phases, namely sample matrix, headspace and fiber coating, across two interfaces (*i.e.*, sample surface/headspace and headspace/SPME fiber coating) [11]. This equilibrium depends on not only the composition of volatile organic compounds but also the matrix effect which influences the volatility of volatile compounds. Due to the differences of volatility, volatile compounds would release from sample matrix into headspace at different rates. On the other hand, selective adsorption and/or absorption happened on the fiber based on the affinity between analytes and polymeric fiber coating [3]. Chemicals with high affinity toward the SPME polymer are concentrated and have higher sensitivity compared to other analytes. In particular, Carboxen/PDMS fiber is a unique coating comprising a mixed carbon phase with small micropores. It extracts analytes via adsorption similar to that for porous polymer coatings as generally described while the unique pore structure of Carboxen enables extraction of all analytes without displacement of lighter analytes [12].

As intermolecular interactions play the most important role in extraction by SPME, a non-linear dependence between the amounts of analytes in food matrices and extracted by the sample is expected. The number of sites on fiber surface is limited for adsorption, which resulting in various distribution coefficients between analytes due to competition. As a result, the repeatability of HS-SPME measurement is easily affected by extraction parameters and sample matrix. Therefore, optimization of the extraction parameters is done to improve the quantitative determination of volatiles [13]. Many research works have evaluated the extraction condition and attempted to increase the extraction efficiency and consistency through different approaches, for example, response surface methodology [14], chemometric data treatment via partial least squares model [15], kinetic modeling [16] and improvement of fiber coating [17]. For instance, the regression models used in Ma and Hamid’s research [14] showed a good correlation between measured and predicted data of volatile compounds in cooked beef, including 24 aldehydes, ketones, furans, alkanes, ketones and alcohols, with relatively high R^2^ values, ranging from 0.8455 to 0.9740.

In our previous studies characterizing kinetic models of flavor molecules released from the model foods of chewing gum [18] and alcoholic beverages [19], the characteristic flavor molecules were mainly esters which are relatively small and light molecules. These compounds are highly sensitive with lower boiling point and high aroma activity. The quantification of these molecules is also strongly influenced by extraction parameters, resulting in low repeatability [20]. Through the studies, kinetic models of the targeted molecules were developed based on their order of release, *i.e.*, zero-, first- and second-order. It was proven that the combination of HS-SPME/GC-MS/FID analysis and mathematical modeling could provide more reproducible information compared to the direct measurement of flavor compounds by the HS-SPME/GC-MS/FID method alone. However, among the kinetic models, the quality of the second-order model was yet optimal in term of the variation of model parameters and the model fitness to experimental values. It is therefore necessary to build a new second-order model with improved reproducibility of its kinetic parameters in comparison to that of the experimental results.

This paper focuses on the use of mathematical modeling to optimize the HS-SPME analysis outcome of second-order flavor release kinetics. In this study, a new model was developed to describe the adsorption and release behavior, achieving high reproducibility of model parameters. The model had a root in describing the amount of intermediate products in a chemical reactor with parallel first-order reactions [21] as well as psychological modeling such as the anxiety information equation [22]. By a two-loop optimization process that minimized the relative standard deviations (RSD) of model parameters and the root mean square deviation (RMSE) of model fitness, the model was able to describe the release profiles of different analysts and conditions with high performance. This model together with the models reported in our previous studies [18,19], once established, could provide a systematic description of the SPME fiber behavior regarding wide range of low molecular weight compounds, which could be applied to many fields, such as environmental, pharmaceutical and flavor analysis with similar kinetics or dynamics.

## 2. Methodology

### 2.1. Principle of Existing Model

In our previous studies [18,19], a second-order kinetic model was developed by solving a secondary ordinary differential equation:

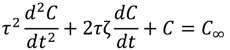
(1)
where *C* and *C_∞_* were the concentration at time *t* and equilibrium concentration of an analyte on the fiber, respectively. It describes the release pattern where a drop in analytes’ concentration occurs after reaching its maximum. Such a drop might be due to the competition for limited space on the ﬁber with other ﬂavor compounds that had stronger afﬁnities to the ﬁber. Two kinetic constants (*i.e.*, τ and ζ) were defined to characterize the behavior of release, where τ represented the natural period or the extraction’s dynamic response speed, and that ζ was the damping factor of the process. Through mathematical modeling, the RSD of the model parameters were reduced. However, it was found that compared to zero- and first-order release models, the RSD values, especially those of ζ, were large and the fitness of predicted values to experimental results was poorer due to the oscillation nature of the model [18,19,23].

### 2.2. Principle of New Model

A new second-order kinetic model was developed to describe the dynamic balance between the adsorption amount of a flavor compound in the headspace onto the SPME fiber and the amount of the compound released from the fiber to the headspace. It was modeled as a combined effect of two parallel first-order reactions, namely the release kinetics and the adsorption kinetics of volatile compounds onto the fiber, as follows:

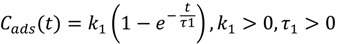
(2)

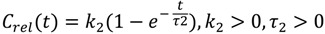
(3)
where *C_ads_*(*t*) and *C_rel_*(*t*) are the concentrations of a flavor compound due to adsorption and release, respectively, on the fiber at time *t*. *k*_1_ and τ_1_ are model parameters of adsorption, while k_2_ and τ_2_ are model parameters of release. *k*_1_ and *k*_2_ are the equilibrium constants while τ_1_ and τ_2_ are time constants of the reactions. In addition, *C_∞_* is the equilibrium concentration of the flavor compound on the fiber after a prolonged period. 

Subtracting Equation (3) from Equation (2) yields the actual amount of the compound on the fiber, where *C*(*t*) is defined as the concentration of analyst on the fiber at time *t*:

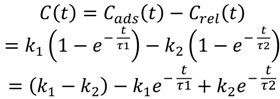
(4)


Therefore, the concentration of an analyte on the fiber can be described as a function of time, involving four model parameters, *i.e.*, *k*_1_, *k*_2_, τ_1_ and τ_2_. The maximum concentration *C_max_*, time at the maximum concentration *t_max_* and the equilibrium concentration *C_∞_* can be derived from Equation (4), as follows:

*C_∞_=k*_1_ − *k*_2_ (5)

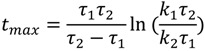
(6)

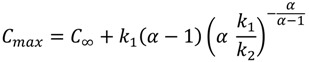
(7)
where 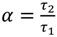 and τ_1_ < τ_2_.

### 2.3. Modeling Data

In the model system of chewing gum, sugar-base chewing gum samples were made up of fine icing sugar, glucose syrup, gum base, glycerin and flavoring, while the sugar-free chewing gum samples were made up of sorbitol powder, xylitol, sorbitol syrup, gum base, aspartame, mannitol, glycerine and flavoring. The flavorings consisted of 33 selected flavoring compounds dissolved in vegetable oil. On the other hand, alcoholic beverage samples consisted of 40% v/v ethanol and 0.62% v/v flavoring that was prepared by dissolving selected 10 esters in 100 mL ethanol. The experiments were conducted by a combined technique of HS-SPME and GC–MS/FID. The release profile of each compound was measured in triplicate, recording peak area changes against SPME extraction time. The extraction-time profiles were examined at three different temperatures (*i.e.*, 30, 40 and 60 °C) of which the first two corresponded to the typical storage condition (*i.e.*, room temperature) and approximate consumption temperature (*i.e.*, mouth temperature), respectively. Furthermore, 60 °C was selected as a typical processing temperature of chewing gum, and for a better knowledge of the kinetic release behavior of less volatile flavor compounds in alcohol beverages. From the flavor release patterns observed in the alcoholic beverage model system, flavor compounds behaved quite differently at the three designated temperatures. Among them, a number of flavor compounds namely ethyl hexanoate, ethyl decanoate, hexyl acetate, *cis*-3-hexenyl acetate, allyl hexanoate, ethyl octanoate, isoamyl hexanoate, and ethyl lauroate were found to exhibit second-order kinetics. However, poor reproducibility in model fitness (*i.e.*, RSD values of ζ) was observed. With the experimental dataset, the release profiles of these compounds were reexamined in the present study for testing the new kinetic model. From the release profile of the chewing gum model system observed at different environmental temperatures, three compounds (*i.e.*, ethyl butanoate, ethyl hexanoate and isopentyl acetate) exhibited second-order flavor release behavior, and they were also used to fit into the new model. The experimental dataset was normalized first by dividing the peak areas to *C_∞_* of each run.

Derived model parameters (*i.e.*, *k*_1_, *k*_2_, τ_1_, τ_2_ and *W*) were validated using one experimental data point for each of the compounds at *t* = 300 min in the alcoholic beverage and data points at *t* = 20 min in the chewing gum. If the peak time *t_max_* of a certain compound, e.g., isoamyl hexanoate from alcoholic beverages at 30 °C, appeared at the 300th min, another data point would be chosen for this compound.

### 2.4. Modeling Process

The modeling process was implemented through a two-loop optimization procedure using Matlab R2011b (The MathWorks, Inc., Natick, MA, USA). Five model parameters were included in the modeling procedure, namely, *k*_1_, *k*_2_, τ_1_, τ_2_ and *W*. Parameters *k*_1_, *k*_2_, τ_1_, and τ_2_ described the release kinetics of an analyte for each experimental run, while weighting factor *W* indicated the relationship among the triplicate runs. The statistical quantity, RSD was used to describe variations of the model parameters, while RMSE described the overall fitness of the model to the experimental data. 

**Figure 1:**
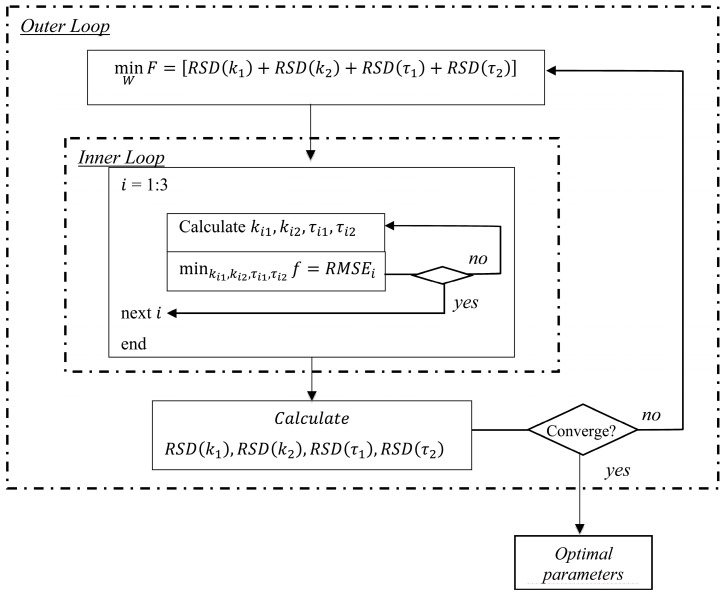
Block diagram of optimization procedure.

The programme consisted of two optimization procedures as illustrated in Figure 1. The inner loop optimized the model fitness to the experimental data, *i.e.*, fitting the experimental data in the flavor release model to derive four kinetic parameters, *k*_1_, *k*_2_, τ_1_, and τ_2_. The inner loop aimed to achieve the lowest RMSE. On the other hand, the outer loop was to find the smallest RSD of each model parameter by adjusting a weighting factor *W*, based on the results from the inner loop. The inner loop yielded four model parameter values, *i.e.*, *k*_1_, *k*_2_, τ_1_ and τ_2_, simultaneously for each experimental run with a set of equations including Equations (4)–(7) and the measured data set. The outer loop optimized *W*, which yielded the smallest sum of RSD of the four model parameters, *i.e.*, the value of *F*.

In both loops, Matlab’s *fmincon* function and the interior-point algorithm were used to adjust the model parameters and the weighting factor. A set of initial values of the four parameters *k*_1_, *k*_2_, τ_1_ and τ_2_, and the weighting factor *W* were assigned at the start of the optimization procedure together with a set of lower and upper bounds. The data points used for validation were not used in the modeling procedure.

## 3. Results and Discussion

### 3.1. Model Performance

**Figure 2:**
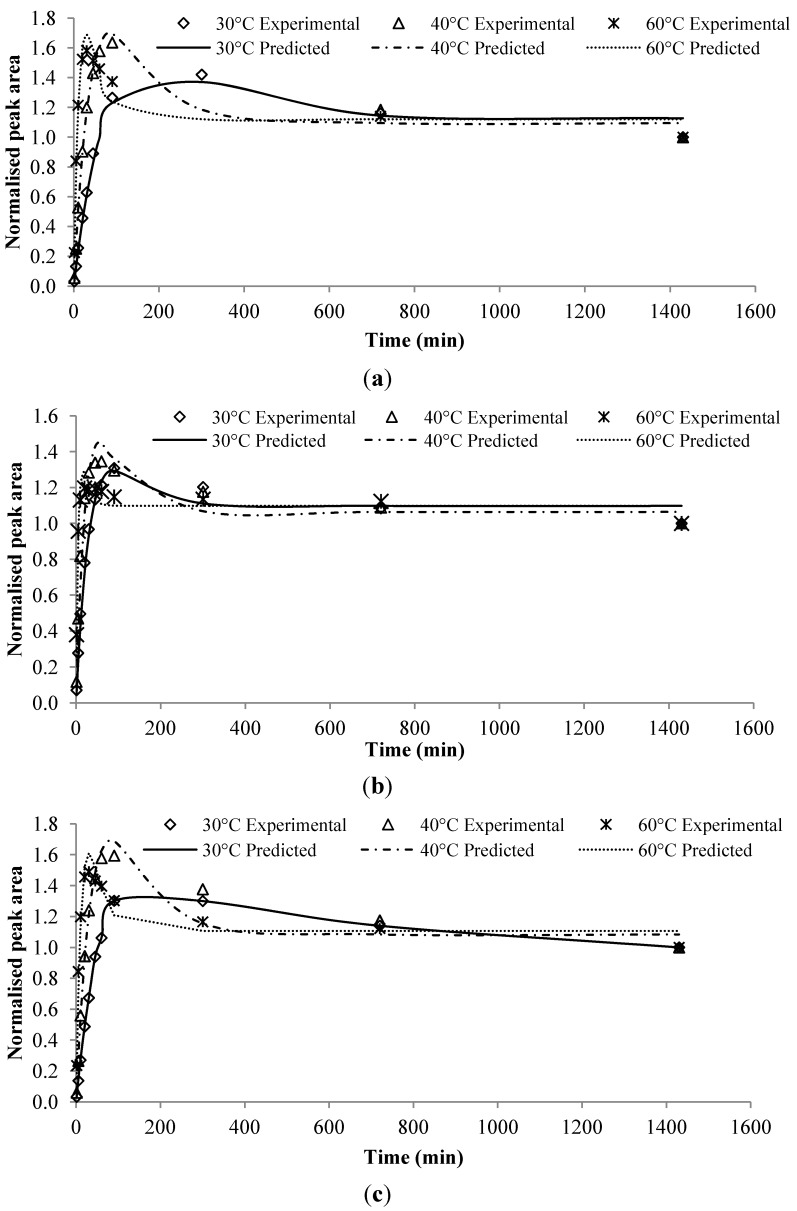
Predicted profile by the second-order kinetic model for flavor release of selected flavor compounds in alcoholic beverage (**a**) isoamylhexanoate; (**b**) allylhexanoate; (**c**) ethyl octanoate. The averaged peak areas are presented as normalized quantities.

The performance of the developed model was demonstrated on seven flavor compounds commonly present in alcoholic beverages, which were known to exhibit second-order kinetics at different temperatures. As the experimental dataset was normalized by dividing the peak areas to *C_∞_* of each run, the predicted modeling results were compared with normalized peak areas. With respect to the normalized peak area, models for isoamyl hexanoate, allyl hexanoate and ethyl octanoate fitted very well to their corresponding experimental data (Figure 2).

Table 1 summarizes the evaluation results and demonstrates the good precision of the developed model based on the RSD values of the four kinetic parameters. Compared to the previous model [19], the RSD values obtained for all of the selected flavor compounds were further reduced.

**Table 1:** Summary of weighting factor, RSD of parameters and RMSE of triplicates for flavor compounds in alcoholic beverage and chewing gum.

Model Food Systems	*W*	F (%)	k_1_		k_2_		τ_1_		τ_2_		RMSE
			Average	RSD (%)	Average	RSD (%)	Average	RSD (%)	Average	RSD (%)	
**Alcoholic Beverage**											
**30 °C**											
Ethyl Hexanoate	5.95	13.48	3.11 × 10^9^	2.41	3.10 × 10^9^	2.36	13.34	4.33	13.50	4.37	0.17
Hexyl Acetate	326.38	15.33	1.77 × 10^9^	3.62	1.75 × 10^9^	3.52	21.93	4.15	22.44	4.04	0.10
Cis-3-Hexenyl Acetate	199.50	7.16	4.06 × 10^9^	3.54	4.04 × 10^9^	3.56	22.00	0.00	22.09	0.05	0.08
Allyl Hexanoate	1.15	3.61	6.23 × 10^9^	1.09	6.18 × 10^9^	1.03	47.96	0.77	48.40	0.72	0.09
Ethyl Octanoate	304.83	8.89	6.93 × 10^9^	2.36	6.85 × 10^9^	2.26	106.94	2.39	108.82	1.87	0.06
Isoamyl Hexanoate	349.99	7.52	9.27 × 10^9^	3.58	9.17 × 10^9^	3.65	113.10	0.29	115.00	0.00	0.05
**40 °C**											
Ethyl Hexanoate	3.53	2.55	2.58 × 10^9^	0.36	2.57 × 10^9^	0.35	13.63	0.87	13.80	0.98	0.19
Hexyl Acetate	885.24	12.72	1.67 × 10^9^	0.81	1.65 × 10^9^	0.92	11.72	5.49	11.90	5.51	0.06
Cis-3-Hexenyl Acetate	28.86	10.69	8.30 × 10^8^	3.11	8.18 × 10^8^	3.28	14.17	1.99	14.37	2.30	0.12
Allyl Hexanoate	30.88	5.84	3.01 × 10^9^	0.73	2.98 × 10^9^	0.67	35.79	2.16	36.53	2.28	0.12
Ethyl Octanoate	227.48	5.94	8.71 × 10^9^	2.55	8.66 × 10^9^	2.52	58.30	0.46	59.08	0.41	0.11
Isoamyl Hexanoate	34.02	4.85	8.01 × 10^9^	0.17	7.94 × 10^9^	0.23	61.86	2.20	63.09	2.25	0.10
Ethyl Decanoate	863.01	16.56	2.25 × 10^10^	3.56	2.23 × 10^1^^0^	3.72	322.53	4.71	326.39	4.57	0.04
**60 °C**											
Allyl Hexanoate	748.05	4.69	1.38 × 10^9^	2.36	1.36 × 10^9^	2.31	9.10	0.00	9.26	0.02	0.07
Ethyl Octanoate	2.00	7.74	2.67 × 10^9^	1.16	2.64 × 10^9^	1.18	19.82	2.58	20.26	2.81	0.09
Isoamyl Hexanoate	2.81	6.55	3.51 × 10^9^	1.34	3.47 × 10^9^	1.37	20.27	1.82	20.77	2.02	0.08
Ethyl Decanoate	241.21	13.48	8.10 × 10^9^	4.85	8.02 × 10^9^	4.82	73.81	1.90	75.34	1.90	0.04
Ethyl Lauroate	642.31	13.42	6.10 × 10^10^	2.21	6.08 × 10^10^	2.28	358.69	4.51	360.80	4.42	0.06
**Chewing Gum at 60 °C**											
Sugar-free ethyl butanoate	37.94	3.28	1.34 × 10^10^	1.05	1.33 × 10^10^	0.99	6.17	0.67	6.23	0.56	0.09
Sugar-base ethyl butanoate	1.01	5.10	3.68 × 10^9^	2.18	3.63 × 10^9^	2.06	2.49	0.27	2.51	0.58	0.04
Sugar-free ethyl hexanoate	8.51	2.88	2.81 × 10^10^	0.26	2.77 × 10^10^	0.34	9.71	1.11	9.84	1.17	0.10
Sugar-base ethyl hexanoate	11.38	8.31	2.18 × 10^10^	0.57	2.16 × 10^10^	0.58	4.29	3.57	4.32	3.58	0.07
Sugar-free isopentyl acetate	13.00	5.68	3.40 × 10^9^	0.37	3.36 × 10^9^	0.38	6.11	2.28	6.28	2.65	0.13
Sugar-base isopentyl acetate	81.08	13.33	1.86 × 10^9^	2.28	1.85 × 10^9^	2.34	2.05	4.36	2.10	4.34	0.08

All of the RSD values were less than 5%, except for hexyl acetate at 40 °C in the alcoholic beverage model system (for which the RSD values of τ_1_ and τ_2_ were at 5.49% and 5.50%, respectively), indicating a satisfactory reproducibility of the characterization results. In addition, the total RMSE of each compound was relatively low, with most of the values being less than 0.15. It indicated that the average RMSE for each measurement data set was less than 0.05 and therefore the model had a good fitting to the experimental data. 

Regarding the previous second-order kinetic model 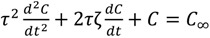 [18,19], the model yielded high RSDs for some of the flavor compounds in the alcoholic beverage system. The RSDs of τ and ζ values in the previous model ranged from 0% to 5.7% and 3.6% to 63.3%, respectively. This reflected the poor reproducibility of ζ values due to large variations in the amplitude of the overshoot between *C_max_* and *C_∞_* in the experimental replicates. In the plot of model predicted values *vs.* experimental values, data points were scattered around the diagonal line in low concentration range illustrating a good fitting in the range; however, notably a clear trend of becoming horizontal at high experimental concentration range was observed (data not shown).

To improve the fitness of the model to the experimental results and reduce the RSD values of the model parameters at the same time, a weighting factor W was introduced in the newly developed model. Empirical guidelines for choosing the initial values of τ_1_ and τ_2_ (Table 2) were also developed to facilitate the inner loop of the optimization procedure. As a result, the new model and the corresponding kinetic parameters, which provide a measure of the combined effect of a release kinetics and an adsorption kinetics onto the fiber, can provide more reproducible information on the overall release profile of those volatile compounds of second-order release behavior, compared to the direct measurement of the compounds by using the HS-SPME/GC-MS/FID method alone.

**Table 2:** Selection rules for the initial values of τ_1_ and τ_2_, depending on the value of *t_max_*.

*t_max_* (min)	τ_1_ and τ_2_ *
3–5	1–3
10	4–9
20	9–14
30	13–22
45	20–30
60	30–40
90	45–80
300–720	100–370

* Generally applicable when the initial values assigned to *k*_1_ and *k*_2_ were between 50–200.

### 3.2. Compromise between RMSE and RSD: Role of Weighting Factor and Prediction of Initial Model Parameter Values

Two statistical quantities were used to evaluate the modeling results, of which RSD described the variations of the model parameters and RMSE described the fitness of the model to the experimental data. Therefore, a compromise between small RMSE and small RSD implies a balance between good fitness to characteristic data points (e.g., the peak point) and that to the rest experimental data points, in order to get the smallest RSD of the parameters. It was especially a concern when the triplicate experimental data differed a lot from each other, e.g., when the RSD of peak points was larger than 10%, or when *t_max_* or *C_max_* differed significantly within the triplicates. Therefore, when the triplicate data sets deviated too much from each other, minimizing the RMSE would lead to a large RSD of the parameters, and vice versa. 

The weighting factor *W* was introduced to the optimization procedure, enabling the procedure to automatically achieve an optimized result, *i.e.*, a balance between the smallest RMSE and the smallest RSD. The weighting factor *W* decided the fitting priority to either the characteristic data point (*t_max_*, *C_max_*) or the rest of the measurement data points. The differences among the three data sets (*i.e.,* experimental triplicates) were taken into consideration, and the weighting factor was adjusted to avoid over-fitting to the point of (*t_max_*, *C_max_*) or the rest of the measurement data points. As a result, *W* was able to minimize the RMSE and RSD values to an acceptable range, even when the three triplicate measurement data sets had large variations. A large value of *W* would be expected when *t_max_* or *C_max_* differed greatly among the three data sets. For instance, the release profiles of allyl hexanoate at 40 °C in the alcoholic beverage model system had all three *C_max_* values appearing at *t* = 60 min. The optimized weighting factor *W* was at 30.88, with *F* = 5.84% and RMSE = 0.21. Under the same experimental condition, ethyl decanoate profiles had their *C_max_* appearing at significantly different times among the triplicates. The optimized weighting factor was at 863.00, which was much higher than that of allyl hexanoate. The corresponding *F* value was 16.56%, and RMSE was 0.04, which were of similar scale to those for allyl hexanoate. For allyl hexanoate at 60 °C, *C_max_* appeared at three significantly different times, and the optimized weighting factor became 748.05, with *F* = 4.69%, and RMSE = 0.07.

On the other hand, *W* could be large when the data points prior to or after the peak deviated greatly from each other, even though *t_max_* of the three sets of measurement data were the same. For instance, ethyl decanoate had an optimized weighting factor of 241.21 even though all of its three peak values appeared at *t* = 90 min. The large *W* value was yielded from the large deviation of the data points prior to the peak (more than 10%). Due to the large *W* value resulted from the optimization process, the fitness of the data points prior to the peak point was improved. The RMSE of this compound was 0.04 which was the lowest among all the compounds at 60 °C. As the values of τ_1_ and τ_2_ changed with *t_max_*, the peak of some compounds appeared at significantly different *t_max_* among the three measurement data sets, e.g., ethyl octanoate at 40 °C had the peak at *t* = 90 min twice and at *t* = 60 min once. The modeling results of τ_1_ and τ_2_ were within 50–53, lying at the lower part of the predicted range for the initial values of τ_1_ and τ_2_ when *t* = 90 min as shown in Table 2. Similar results were observed in hexyl acetate and cis-3-hexenyl acetate at 40 °C, where the τ values lied in the range of *t_max_* of two profiles among the three replicates. It implied that the third data set with a different *t_max_* needed to compromise by adjusting the weighting factor or accepting a relatively large RMSE. In general, data with smaller *t_max_* would have smaller τ_1_ and τ_2_. Based on the modeling results, a guideline for selecting the initial values of τ_1_ and τ_2_ was derived and is shown in Table 2.

### 3.3. Validation of the Developed Model

**Figure 3:**
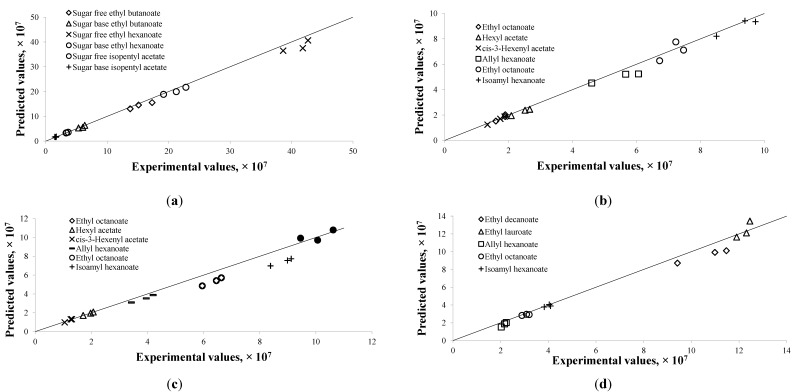
Predicted results *versus* experimental results for flavor compounds in different food models or processing conditions at designated extraction time. For each compound, the triplicates of Y1, Y2, and Y3 are shown. (**a**) Chewing gum at 60 °C; (**b**) Alcoholic beverage at 30 °C; (**c**) Alcoholic beverage at 40 °C; (**d**) Alcoholic beverage at 60 °C.

Given the estimated parameters from the modeling and optimization procedure, the predicted chromatogram peak area of each compound was compared to the experimental peak area at those validation data points described in Section 2.3. These experimental data points were not used in the modeling procedure. The predicted values were very close to the experimental data, as shown in Figure 3. The discrepancies between them were largely within ± 10%, except for few compounds. Large discrepancies were observed in the alcoholic beverage model for ethyl octanoate at 40 °C and isoamyl hexanoate at 40 °C (Figure 3c), which were as high as 18%. The flavor release profiles for both compounds indicated that the equilibrium might not have been reached at the end of the experiment period. Due to that the model assumed the last experimental point as the equilibrium point, the validated results at *t* = 300 min were therefore higher than the corresponding experimental results.

### 3.4. Adsorption and Release of Targeted Flavor Molecules on the SPME Fiber

The newly developed second-order kinetic model described an overshoot scenario, which arises from two competing dynamics that operate on different time scales. This model was designed as a net effect of two parallel, counter-directional first-order reactions, namely, release and adsorption of each analytes on the fiber (Equation (4)). The faster effect of volatile compounds adsorption on the fiber contributed to the initial increase in peak areas, while the slower effect of compounds release from the fiber contributed to the decreases in peak areas. This overshoot response occurred when the initial response, which was adsorption onto the fiber described by Equation (2), was in one direction, while the release effect was in the opposite direction as described by Equation (3) on another time scale. As adsorption is a competitive process, the amount of analytes adsorbed on the fiber depended on its diffusion coefficient in the coating, and the molecules would release back into the headspace when the fiber polymer is attached to a new molecule with higher affinity or kinetic mobility. On the other hand, partition coefficient between matrices and fiber resulted in an equilibrium partitioning process between matrices, headspace and fiber coating. The second-order profile was consisted of the initial increase in the analyte concentration, followed by overshoot and equilibrium at the end. It indicated that adsorption and partitioning happened simultaneously, yet their impact on the fiber changed with time.

Each of the first-order reactions was described by a pair of parameters *k* and τ. The parameter *k* was the equilibrium constant for each first order reaction, indicating the point when the release or adsorption reached equilibrium, while τ was the time constant, indicating the speed of release or adsorption on the fiber. At higher temperature, τ became smaller (as shown in Table 1), which corresponded to the increasing slope of each first order reaction. At higher temperature, the intermolecular activity between analytes in the headspace and polymers on fiber was enhanced, resulting in smaller τ.

**Figure 4:**
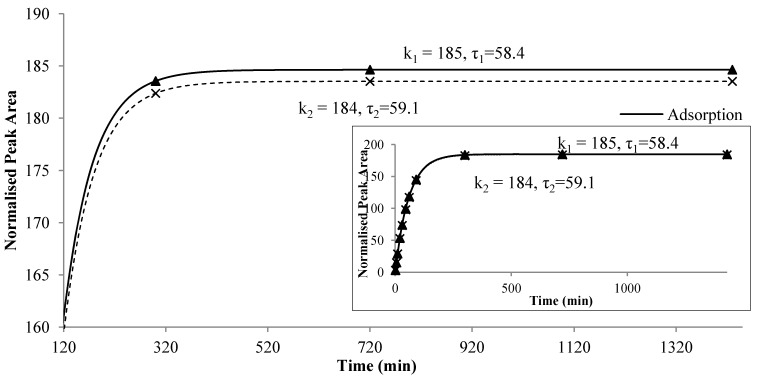
Two first-order reactions, *i.e.*, adsorption onto and release from the fiber of ethyl octanoate in alcohol beverage model system at 40 °C.

Figure 4 illustrates the two reactions for ethyl octanoate in the alcoholic beverage system at 40 °C. The increasing slope of each first-order reactions is related to τ_1_ and τ_2_, respectively. The larger the value of τ, the smaller was the slope, reflecting a slower reaction. At the beginning, the adsorption was faster than the release which had a smaller model parameter τ_2_. As the time proceeded, the rate of increasing decreased and a maximum difference between the two reaction curves appeared at a certain point, *i.e.*, *C_max_*. After that time, the adsorption and the release were quickly approaching the equilibrium and the amount of compound remained on the fiber was almost a constant. Furthermore, the values of *k* and τ depended on the compound, food matrix and environmental temperature. The difference between τ_1_ and τ_2_ also varied among the compounds. The value of weighting factor *W* may reflect the instrumental uncertainties during experiment. Therefore, the new model, together with its parameters, might provide more insight to the nature of flavor release, especially the dynamic balance between adsorption and release of flavor compounds onto the fiber.

## 4. Conclusions

The novel approach of combining HS-SPME/GC-MS and mathematical modeling has been proved to be able to improve the reproducibility of the release behavior characterization of flavor compounds from food matrices that were analysed through HS-SPME. Through mathematical modeling, the model parameters were shown to perform better and be more reproducible in describing flavor release in both semi-solid and liquid food systems, *i.e.*, chewing gum and alcoholic beverages. In the current study, a significant improvement was achieved by developing a new second-order model through a two-loop optimization procedure, which yielded both reproducible results and better fitness of the model to the experimental data. Furthermore, the model provided much more insights to the release mechanisms of the flavor compounds, which could have significantly different behaviors at various environmental temperatures and in different food matrices.
